# Exploring the origin and potential for spread of the 2013 dengue outbreak in Luanda, Angola

**DOI:** 10.3402/gha.v6i0.21822

**Published:** 2013-08-02

**Authors:** October M. Sessions, Kamran Khan, Yan'an Hou, Eyal Meltzer, Mikkel Quam, Eli Schwartz, Duane J. Gubler, Annelies Wilder-Smith

**Affiliations:** 1 Program in Emerging Infectious Diseases, Duke-NUS, Singapore; 2 Division of Infectious Diseases, St. Michael's Hospital, University of Toronto, Toronto, Ontario, Canada; 3 Center for Geographic Medicine and Department of Medicine C, Sheba Medical Center, Tel Hashomer, Israel; 4 Sackler Faculty of Medicine, Tel Aviv University, Israel; 5 Institute of Public Health, University of Heidelberg, Heidelberg, Germany; 6 Lee Kong Chian School of Medicine, Nanyang Technological University, Singapore; 7 Epidemiology and Global Health, University of Umea, Umea, Sweden

**Keywords:** dengue, Angola, dengue sequence, international travel, dengue in Africa

## Abstract

**Introduction:**

Dengue in Africa is underreported. Simultaneous reports of travellers with dengue returning from Luanda, Angola, to six countries on four continents suggest that a major dengue outbreak is currently occurring in Angola, South West Africa.

**Methods:**

To identify the origin of the imported dengue virus, we sequenced the virus from Angola and investigated the interconnectivity via air travel between dengue-endemic countries and Angola.

**Results and Conclusion:**

Our analyses show that the Angola outbreak was most likely caused by an endemic virus strain that had been circulating in West Africa for many years. We also show that Portugal and South Africa are most likely at the highest risk of importation of dengue from Angola due to the large number of air passengers between Angola and these countries.

Dengue viruses (DENVs) are the world's foremost arboviral disease, affecting an estimated 400 million people annually and with over 50% of the world's population living in regions at risk of contracting the disease (1, 2). Over the past 50 years, the geographic distribution and incidence rate of DENV infection has exploded, likely due to virus evolution, deteriorating vector control, the increase in international travel and uncontrolled urban growth (2–5). However, the epidemiology of DENV in sub-Saharan Africa is poorly understood and the disease is likely grossly under-reported due to low awareness of DENV among health care providers in Africa, lack of diagnostic testing, and poor surveillance (6). Travellers may act as sentinels to uncover ongoing outbreaks not reported in Africa (6, 7).

In early May 2013, the global GeoSentinel network reported 10 cases of dengue fever, and Portugal reported 19 cases in travellers returning from Luanda, Angola, South-West Africa (8). The capital city of Luanda is Angola's largest seaport, with an estimated population of 5–20 million people (9). DENV cases in Angola have been described in the past, but not in the recent years (6). The simultaneous reports of travellers with DENV to six countries on four continents suggested that a major DENV epidemic was occurring in Angola. Indeed, after the initial alert through findings from international travellers, more accurate data have emerged. As of 31 May 2013, a total of 517 suspected DENV cases had been reported and 313 specimens tested positive for DENV in Angola, including one from a patient who died (9).

The origin of the DENV-1 virus causing the outbreak in Angola is of interest. Complete DENV genome sequencing is essential to this understanding and can also aid in tracking the spread of the virus to other susceptible areas (10). Furthermore, information on air traffic can be used to explore the most likely countries of origin and to predict the risk of further spread to other regions (11).

At least 91 laboratory-confirmed DENV cases have been reported in April–June 2013 in seven countries (Canada, France, Germany, Israel, Portugal, South Africa, and the United States) among persons who had recently travelled to Luanda, Angola (9). In this, we report on the most likely origin of the DENV responsible for the 2013 Angola outbreak based on complete genome sequencing of the DENV-1 isolated from an Israeli traveller returning from Luanda, Angola. We also analyse the interconnectivity via air travel between Angola and other at-risk countries.

## Methods and results

We obtained a blood sample during the acute phase of illness of a polymerase chain reaction (PCR)-confirmed DENV case, an Israeli traveller who had acquired DENV in Luanda, Angola, and returned to Israel reporting to one of the GeoSentinel sites in April 2013 (8). The blood sample was sent for sequencing to the Emerging Infectious Diseases Program at Duke-NUS Graduate Medical School, Singapore. Viral RNA was extracted from serum using a QIAamp Viral RNA Mini Kit (QIAGEN, Valencia, CA, USA) according to the manufacturer's instructions and stored at −80°C until use. A one-step multiplex assay for the detection of the specific serotype of DENV was carried out according to the protocol described by Johnson et al. (12). Upon detection of patient's serum to be of DENV-1, reverse transcription to yield cDNA was performed using Invitrogen Superscript III First Strand Synthesis System (Life Technologies, Carlsbad, CA, USA), according to the manufacturer's instructions using 10 µM gene-specific reverse primer D1-F5-10693R (Table 1).

**Table 1:** Primer sequences used to amplify and sequence the Angola DENV1 isolate

	PCR primer name	PCR primer sequence (5' –3')	Sequencing primer	Sequencing primer sequence (5'–3')
Fragment 1	D1-F1-5F	GTTAGTCTACGTGGACCGAC	D1-1F (D1s1C)	*GATGAGGGAAGATGGGG* AGTTGTTAGTCTACGTGGAC
	D1-F1-2084R	CACCTGCTCCTATCACGATG	D1-854F	CTAGCACATGCCATAGGAACATCC
			D1-1260R	CCGAAGAGCCCACAGCCATTGC
			D1-1588F	GCCTTGGACCTCGGGAGCCTC
Fragment 2	D1-F2-2201F (D1s6)	GGYTCTATAGGAGGRGTGTTCAC	D1-3033R (D1a16)	CARCTTCCARGTYTCGTTCTT-
	D1-F2-4561R (D1a13)	TTCCACTTCYGGAGGGCT	D1-3543R (D1a15)	GCATYTTTCTRCTCCATCTGGATC
			D1-4033R (D1a14)	CCGGAAGCCATGTTGTTTT
			D1-3241F (D1s8)	ACAAACAGCAGGGCCRTGGCA
			D1-4543R (D1a13)	TTCCACTTCYGGAGGGCT
			D1-3735F (D1s9)	CCTAGCYYTGATGGCYACTTT
Fragment 3	D1-F3-4221F	CACTAATAGCTGGAGGCATGC	D1-4238R (D1a12)	CCTCGTCCTCAATCTCTGGTAG
	D1-F3-6461R	CCAGGTTGTCCAAGGCATTC	D1-4541R (D1a13)	TTCCACTTCYGGAGGGCT
			D1-4541F	CCTAGCCCTCCAGAAGTGG
			D1-4575R (D1a11)	CRTAGCCTGARTTCCATGATCT
			D1-4742F (D1s11)	AAGAGRCTGGAACCRAGYTGGGC
			D1-5018R (D1a10)	TCTCTCYGGCTCAAAGAGGG
			D1-5243F (D1s12)	AAATGGCAGAGGCGCTCAAGGG
			D1-5575R	GATCCACTCATAGCCTGAGTTCC
Fragment 4	D1-F4-6442F	GAATGCCTTGGACAACCTGG	D1-6537F (D1s15)	GGATAGCGGCCTCYATCATACT
	D1-F4-8519R	CACCATTGACCATGGATGAGGC	D1-7089R (D1a6)	AGRACACGTAACGTTCTWCCTTC
			D1-7252F (D1s16)	GCAAARGCYACTAGAGAAGCTCAA
			D1-7537R	GGCCAGACCTGCTCCTGCTAG
			D1-7921F	GGCGACCTATGGATGGAACC
			D1-8558F (D1s18)	CCACYCATGAAATGTAYTGGGT
Fragment 5	D1-F5-8540F	GCCTCATCCATGGTCAATGGTG	D1-8542R (D1a18)	AAAGGTGGYTCYGYYTCAAT
	D1-F5-10693R	CTGTGCCTGGAATGATGCTG	D1-9613R	CAGAGCTGTTAAGGCTGTTGC
			D1-8750F (D1s19)	GCCARGTGGTTATGGGGTTT
			D1-8994F	GGTACATGTGGTTGGGAGCACGC
			D1-9620F (D1s20)	GGATGATCTTCAGAATGAGGC

The DENV-1 genome was amplified in five fragments with the following primer pairs: D1-F1-5F and D1-F1-2084R, D1-F2-2201F and D1-F2-4561R, D1-F3-4221F and D1-F3-6461R, D1-F4-6442F and D1-F4-8519R, D1-F5-8540F and D1-F5-10693R (Table 1). One microlitre (µL) of cDNA was mixed with 1 µL of each primer (10 µM) and 17 µL of Intron i*pfu* Mastermix. The PCR cycling conditions were: 94°C for 60 s followed by 40 cycles of PCR at 94°C for 10 s, 50°C for 10, s and 72°C for 2 min and a final extension at 72°C for 2 min. All PCR fragments were excised from a 1% preparative agarose gel and extracted with the Qiagen Gel Extraction Kit. PCR products were sequenced with its respective forward and reverse primers used for amplification as well as internal sequencing primers sitting within the PCR fragment (Table 1).

**Fig. 1:**
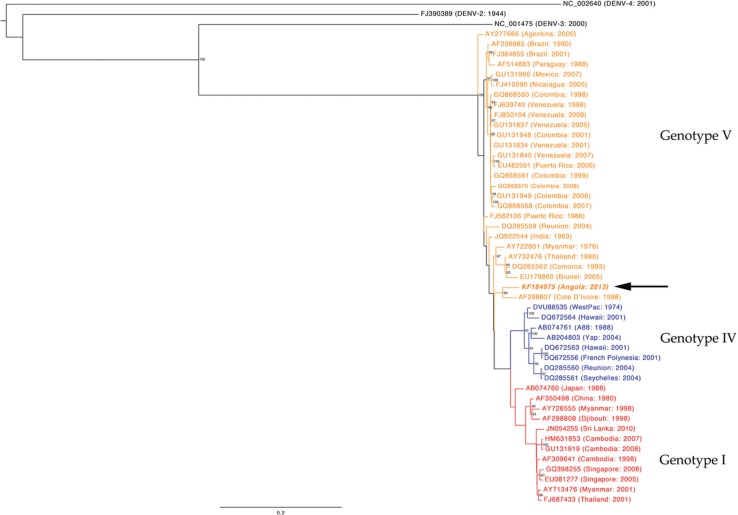
Phylogenetic analysis of the Angola DENV-1. DENV-1 nucleotide sequence from the Angola outbreak was aligned with representative DENV-1 sequences from around the world representing multiple genotypes. Black-coloured isolates are representative of DENV2-4 serotypes. Blue-coloured isolates represent genotype IV, red-coloured isolates represent genotype I and orange-coloured isolates represent genotype V. The DENV-1 sequence from Angola (AF298807) is depicted in bold-italics.

Multiple sequence alignment of the Angola DENV-1 complete genome to other DENV-1 sequences deposited in GenBank was carried out using a fast Fourier transform in MAFFT (13). The maximum-likelihood phylogenetic tree was inferred from the sequence alignment using RAxML (14, 15). The robustness of the maximum-likelihood tree was assessed by 1,000 maximum-likelihood bootstrap replications. The maximum-likelihood tree was visualised and produced using FigTree v1.4.0 (16) (Fig. 1).

**Fig. 2:**
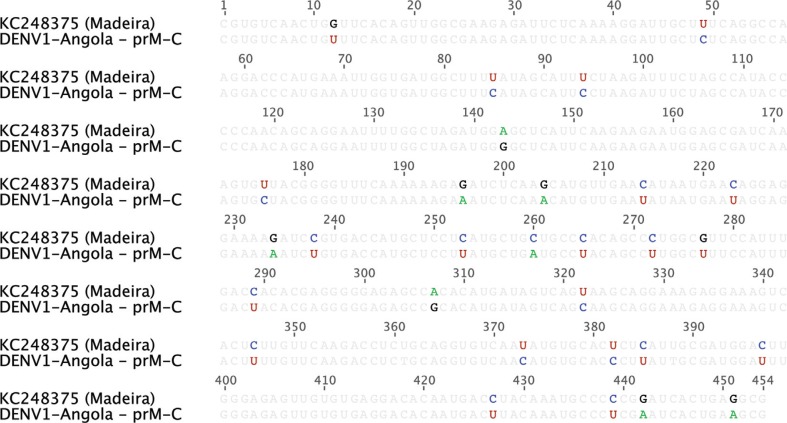
Alignment of the Angola DENV-1 and the Madeira DENV-1 sequences. The prM-C portions of the Angola and Madeira DENV-1 sequences were aligned. Highlighted bases indicate sequence differences.

Phylogenetic analysis of the full DENV-1 genome sequence shows that the Angola DENV-1 belongs to genotype V (Fig. 1) and is most closely related (94.7% nucleotide sequence identity) to the Cote D'Ivoire, Abidjan virus isolated from a French soldier in 1998 (17). In addition, we directly compared our Angola DENV-1 sequence with the available sequence data from South America and the recent novel Madeira outbreak (Fig. 2) (18). The analysis shows that these sequences share 92.8–94% and 93.6% identity, respectively.

**Fig. 3:**
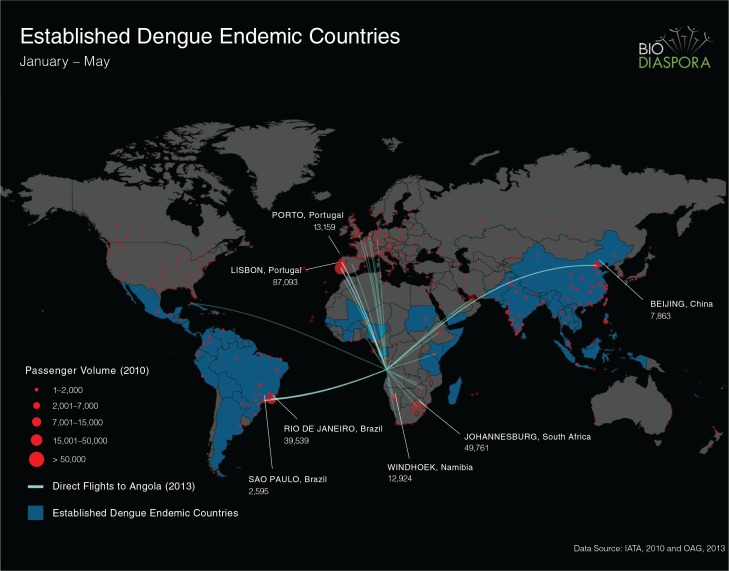
Air passenger numbers and direct flights from Luanda, Angola.

To describe global air travel patterns to Luanda, Angola, we analysed worldwide passenger-level flight itinerary data from the International Air Transport Association between January and May 2012, taking into consideration all traveller flight connections (direct and indirect) to determine the initial origin and final destination of each traveller. Furthermore, we identified all direct flights to Luanda from January to May 2013 using data from the Official Airline Guide. We created a map that depicts the direct routes into Angola from dengue-endemic countries (lines) and the number of air passengers who initiated travel from cities worldwide with a final destination in Angola (by the size of red dots) (Fig. 3). Although not shown, the global distribution of flights and travellers departing Angola was highly symmetric to observed patterns of inbound travel.

The flight data analyses showed that of all the DENV-endemic countries, Brazil had by far the highest number of air passengers to Angola, followed by China (not highly DENV-endemic), Congo (Brazzaville), India, Philippines, Ethiopia, and Cape Verde (Table 2). There are 71 direct flights departing from Angola. In terms of air passenger numbers from Angola to non-DENV-endemic countries, Portugal and South Africa are the leading destinations (Fig. 3).

**Table 2:** Numbers of air travellers departing dengue-endemic countries with a final destination in Angola, January–May 2012

Dengue-endemic country	Passenger numbers
Brazil	42,835
China	11,824
Congo(Brazzaville)	6,521
India	5,012
Philippines	3,833
Ethiopia	2,283
Cape Verde	1,470
Thailand	556
Pakistan	408
Indonesia	336
Nigeria	285
Argentina	222
Singapore	194
Australia	182
Malaysia	143
Ghana	81
Mexico	74
Venezuela	73
Colombia	56
Honduras	52
Cameroon	50
Cote D'Ivoire	9

## Discussion

Sequencing data suggest that the currently circulating Angola strain of DENV-1 is most closely related to a DENV-1 isolated in 1998 in Cote d'Ivoire, West Africa (17) (Fig. 1). Thus, it is likely that the Angola outbreak was caused by a virus that has circulated in West Africa for years, as it is not uncommon for a virus of the same serotype and genotype to circulate in an area for 10–20 years (19, 20). This conclusion is consistent with other sequencing data for DENV-1's isolated from the current Angolan epidemic (21). Although other DENV serotypes have been documented to circulate in West Africa over the last four decades (22), data on DENV-1 are sparse; most likely due to the fact that dengue fever is greatly underreported in Africa (6). This lack of reporting leads to a dearth of available sequence information for Africa as a whole and complicates accurate interpretation of the phylogenetic relationships. In terms of interconnectivity via air routes, air passenger numbers from Cote d'Ivoire to Angola are far smaller compared to other DENV-endemic countries. However, this may not play an important role if this virus has been circulating in West Africa for longer than a decade; the virus may have spread from surrounding regions to Angola via land routes.

As genotype V includes the majority of circulating DENV-1 in South and Central America, another possible explanation may be that there was a more recent introduction from South America (GU131834 and GU131837) or from India (JQ922544) which all share 94% identity with the circulating Angola strain. Brazil reports the highest number of originating air passengers whose flight destination is Angola; India ranks fourth highest in this regard (Table 2). Additional sequence data from these regions, West Africa in particular, are necessary to make a definitive conclusion about the true origin of this DENV-1 strain.

The relative temporal and spatial proximity of the Angola and Madeira (18) DENV-1 outbreaks make it plausible that these events might be directly linked. However, given the overall genetic stability of DENVs (23) and the fact that they only share 93.6% identity, it is most likely that these two outbreaks are separate outbreaks due to independent introductions.

Passenger-level flight itinerary data can also be utilised as a predictor of the most likely places the virus may spread. The high degree of interconnectivity between Angola and Portugal as well as South Africa, suggests that these two countries have an elevated risk of DENV introduction. Indeed, Portugal has reported the highest numbers of DENV cases (79 out of 91; 86%) (9) introduced from Angola, consistent with our flight data. Although *Aedes aegypti*, the main vector for DENV, has not yet been reported in Portugal, that country's close political affiliations with Angola and Madeira suggest that Portugal is potentially at a high risk for spread of DENV from Angola, and thus transmission should the vector be introduced. *Aedes albopictus*, the secondary vector for dengue, is already widespread in Southern Europe. Based on our travel data analysis, South Africa is also at elevated risk for DENV introduction from Angola. South Africa is geographically very close to Angola, and also serves as the main destination for expatriate medical evacuations from Angola. Furthermore, South Africa is also known to harbour *Aedes aegypti* in sufficient numbers to propagate an epidemic of DENV (24).

In summary, the sequence data are highly suggestive, but not definitive that the 2013 outbreak in Angola originated from West Africa. The available sequence from West Africa in GenBank is very limited. A virus from Cote D'Ivoire, Abidjan isolated from a French soldier in 1998, is the closest relative to the Angola virus and shares 94.7% identity. Other potential origins of the Angola DENV-1 strain include India and South America, with the latter being supported by our travel data. We stress that these alternative scenarios should not be summarily excluded from consideration without more sequence information. The lack of DENV sequence data from Africa highlights the need for more effective DENV surveillance efforts in this continent. More DENV outbreaks are expected in the future in Africa, and they are likely to become an increasingly important burden there as it is now in Asia and South America 6. Air traffic intensity can be used to explore the most likely countries of introduction and to predict the risk of further spread. We postulate that Portugal and South Africa, which are currently non-endemic for dengue, are at elevated risk for the introduction of DENV and hence for new emergence.
